# Operative Treatment of Peritonitis

**Published:** 1893-08

**Authors:** 


OPERATIVE TREATMENT OF PERITONITIS.—A number of articles have recently appeared from the pens of surgeons whose enthusiasm exceeds their discretion, advocating operative interference in all cases of peritonitis, many of these have contained no gratifying claim to the statement: "If the patient is not dying laparotomy is justifiable." It is, therefore, a relief to read an article from an experienced abdominal surgeon condemning the general adoption of this procedure. Dr. Joseph Eastman, of Indianapolis, one of the world's best operators, has such an article in the *Journal of the American Medical Association.* In the course of it he says: In the earlier stages of septic peritonitis (and all inflammation is of septic origin), surgical interference is but adding fuel to the flames. After the patient has been tided over the acute stage of the disease, to the time when the micro-organisms have become less active, surgical efforts may be curative by removal of the localized products of inflammation. Cases have come to my knowledge where practitioners anxious to obtain the prestige presumably gained by abdominal section, have ventured to open the swollen abdomen with no definite idea what they were going after, where they were going to locate it, or what they were going to do with it, after they had found it. After opening the abdomen freely, with the hope that they might see something which the finger had not been able to feel, they found the intestines distended with gas, piled up in formidable stacks over the abdomen and even falling down on the table. Their position was something like the man who attempted to repair the clock; he had no difficulty in taking the clock to pieces, but when he attempted to put it together again he imagined he had wheels enough for fourteen clocks.

